# Role of MRI in evaluation of small bowel disease

**DOI:** 10.1186/1470-7330-15-S1-P5

**Published:** 2015-10-02

**Authors:** Foad Serag El-Dein, Khaled M Moghazy, Amany El-banna, Ahmed Hafez Afifi, Abeer Abo-Ellela

**Affiliations:** 1 Radio-diagnosis Department, Alexandria University, Alexandria, Egypt

## Introduction

Magnetic Resonance Imaging of the Small Bowel (MR Enterography, or MRE) is becoming increasingly popular as the first imaging modality for the diagnosis and follow-up of small bowel diseases. The aim of this work was to evaluate the role of recent MRI sequences and techniques in evaluation of small bowel disease.

## Patients and methods

The study population included 24 patients who were referred to multiple radiology centers by gastroenterologist for magnetic resonance enterography (MRE) for evaluation. The examination was done on 1.5 Tesla superconducting magnet MRI.

## Results

All studied patients had small bowel lesions. 15 patients were neoplastic (64%) and 9 patients were inflammatory (36%). Among 15 cases of small-bowel neoplasm; 12 were malignant; and 3 were benign. The malignant cases were classified as follows; lymphoma (6 patients); adenocarcinoma (4 patients); GIST (1 patients) and carcinoid tumour(1 patient). Nine patients out of 24 had inflammatory bowel diseases. Eight cases out of 9 proved to be specific inflammatory disease and 1 chronic non specific ileocolitis. Seven cases out of 8 proved to be Crohn’s disease and 1 proved pathologically to be TB of small bowel. The final diagnosis was confirmed by surgical or endoscopic data and follow up.

## Conclusions

MRE is accurate non-invasive modalities in assessing the intra-luminal, parietal and extra-luminal small bowel tumour without the need for ionizing radiation. MR signal appearances of the lesions, combined with the contrast enhancement behavior and the characteristic of the stenosis, can help in differentiating neoplastic from other non-neoplastic diseases of small bowel.

